# Hypoglycemia and Factitious Disorders: A Case Report and Review

**DOI:** 10.7759/cureus.26287

**Published:** 2022-06-24

**Authors:** Arya Sharifzadeh

**Affiliations:** 1 Internal Medicine, Florida Atlantic University Charles E. Schmidt College of Medicine, Boca Raton, USA

**Keywords:** hospital based medicine, clinical endocrinology, internal medicine and endocrinology, factitious disorder, sulfonylurea, diabetes mellitus management, nocturnal hypoglycemia, recurrent hypoglycemia, hyperinsulinemic hypoglycemia, severe hypoglycemia

## Abstract

Hypoglycemia may present with a multitude of signs and symptoms ranging from subjective feelings of anxiety or diaphoresis to neuroglycopenic manifestations of altered sensorium or seizure. The differential diagnosis of hypoglycemic disorders is broad, and in rare instances may occur following intentional induction by undisclosed insulin administration or insulin secretagogue ingestion in patients with an underlying factitious disorder. While basic laboratory studies can reliably confirm the presence of exogenous insulin in patients with hyperinsulinemic hypoglycemia, increased endogenous insulin secretion following sulfonylurea ingestion can mimic a biochemical pattern of findings also seen with insulinoma, a rare pancreatic insulin-producing tumor. We present a case of severe hypoglycemia manifesting as diminished consciousness in a patient with multiple medical comorbidities. Following initial laboratory workup suggestive of endogenous hyperinsulinemic hypoglycemia, the results of a serum oral hypoglycemic panel confirmed the presence of glipizide, an unprescribed insulin secretagogue of the sulfonylurea class, in the patient’s serum. In conjunction with psychiatric services, the patient was diagnosed with an underlying factitious disorder and her hypoglycemia was deemed likely the result of surreptitious sulfonylurea ingestion as a pathologic healthcare-seeking behavior. Our case report and subsequent review shed light on critical components in the diagnostic approach to hypoglycemic disorders, which carry significant morbidity for patients regardless of the underlying cause and emphasize several clinical and ethical considerations associated with the identification and management of persons with factitious disorder in medical practice.

## Introduction

A firm understanding of the interaction between medical disease and comorbid psychiatric illness is essential to the everyday practice of patient care. In comparison with simple exaggeration of symptom severity, pathologic healthcare-seeking behaviors of deliberate or malicious deception as seen in factitious disorder (FD) are rare and comprise an uncommon subset of psychiatric diagnoses [1]. While there exists implicit awareness amongst physicians that healthcare-seeking behaviors, although uncommon, may occur in patients of any background, the accurate identification of disease remains of paramount importance and necessitates careful diagnostic workup guided by clinical context. As an example, even a single episode of hypoglycemia severe enough to manifest with neuroglycopenic symptoms obligates a comprehensive stepwise evaluation be undertaken in order to definitively determine the underlying etiology. Hypoglycemia in the absence of diabetes mellitus managed with insulin or insulin secretagogue therapy is uncommon in the general population [2] but is for a small subset of patients with underlying FD among the commonest index presentations to medical providers [3]. When present, FD is therefore a disease entity whose early detection and appropriate management can both limit the overuse of healthcare system resources and ensure no harm may come to patients through unnecessary therapies or invasive procedures.

## Case presentation

A 71-year-old Caucasian female with hypertension, coronary artery disease, major depressive disorder, peptic ulcer disease, and remote history of Roux-en-Y gastric bypass 12 years prior presented to the hospital after an incident of near-syncope during a home physical therapy session. While in the emergency department, the patient became acutely lethargic and was found to have a glucose concentration of 29 mg/dL by capillary blood. Her mentation improved immediately following an ampule of 50% dextrose. In consultation with endocrinology services the following day, the patient described a history of episodic lightheadedness, palpitations, and diaphoresis for several weeks that did not have a temporal association to eating and would awaken her at night. She reported learning to sleep with a glass of orange juice at her bedside because drinking it alleviated her symptoms entirely. She endorsed having been diagnosed with prediabetes 3 months prior to admission but was not taking any medications for glycemic control. She reported home fingerstick glucose readings in the 50 mg/dL range, and stated she was fearful “something is wrong with the pancreas”. She weighed 75 kg with a body mass index of 29 kg/m2. Hemoglobin A1c measured 6.5%. Early morning serum cortisol level and abdominal CT imaging were unrevealing. Several hours after evaluation, a nurse observed the patient becoming acutely confused and disoriented, and measured a glucose concentration of 39 mg/dL by fingerstick capillary blood. Additional testing performed at that time revealed inappropriately elevated levels of plasma C-peptide (4.8 ng/mL; normal range: 0.8-3.5 ng/mL), insulin (35 mIU/mL; normal range: 3-19 mIU/mL), and proinsulin (19.7 pmol/L; normal < 8.0 pmol/L). A qualitative serum sulfonylurea panel was also collected but required shipment to an outside reference laboratory for processing.

After 24 hours without recurrence of spontaneous hypoglycemia, the patient agreed to undergo a supervised 72-hour diagnostic fast. The study was completed without any hypoglycemic episodes. In the final hour of prolonged fasting, plasma glucose concentration measured at 54 mg/dL with a concurrently low C-peptide level (0.6 ng/mL), low-normal serum insulin level (3.0 mIU/mL), and normal proinsulin (3.8 pmol/L) while the patient remained asymptomatic. The rise in plasma glucose concentration was minimal following 1 mg intravenous glucagon administration. Reference laboratory results became available soon thereafter, revealing a positive serum sulfonylurea screen with glipizide levels greater than 5 ng/mL. Although the patient adamantly denied intentional or accidental ingestion of this medication, she was amenable to psychiatric evaluation. The patient later made several conflicting statements regarding her medical and psychiatric history but shared that she had access to medications prescribed to her recently deceased husband including the oral hypoglycemic agent glucotrol, a common brand name for glipizide. She refused an offer to review the contents of a personal handbag in order to ensure no such medication was present. The hospital course concluded uneventfully without recurrence of hypoglycemia. In conjunction with psychiatry services, a diagnosis of hypoglycemia secondary to probable surreptitious sulfonylurea ingestion in the setting of underlying factitious disorder imposed on self was made.

## Discussion

Hypoglycemia is a rare occurrence in the absence of therapeutic insulin or insulin-secretagogue use in persons with known diabetes mellitus [2]. Diagnosis of hypoglycemia requires a plasma glucose concentration low enough to result in compatible signs or symptoms and therefore relies on both biochemical and clinical data. In practice, the presence of a constellation of findings known as Whipple’s triad serves to confirm the diagnosis of a hypoglycemic disorder and determine patients in whom further diagnostic workup for an underlying etiology should proceed. Whipple’s triad requires the presence of symptoms or signs consistent with hypoglycemia, a low plasma glucose concentration, and subsequent symptom resolution after plasma glucose concentration is raised [2]. In the current case, Whipple’s triad was confirmed by objective observation on two occasions during the first 24 hours of hospitalization. Signs and symptoms of hypoglycemia are categorized as neurogenic (e.g. palpitations, tremulousness, diaphoresis, anxiety, etc.), largely a result of physiologic changes induced by autonomic nervous system discharge, or neuroglycopenic (e.g. confusion, altered or diminished consciousness, seizures, etc.), broadly reflecting central glucose deprivation at the level of the brain [2]. Hypoglycemic symptoms are typically absent unless plasma glucose drops below 55 mg/dL in healthy individuals, but this glycemic threshold is approximate and may shift to higher plasma glucose concentrations in persons with diabetes mellitus or lower concentrations in those with recurrent episodes of hypoglycemia [2]. While neurogenic symptoms can occur even in the absence of a critically low plasma glucose concentration, neuroglycopenia is considered a life-threatening complication of hypoglycemia for which meticulous diagnostic workup, as in the current case, is compulsory.

Factitious disorder (FD) is also a rare diagnosis when compared with other psychiatric conditions [1, 3]. Accurate identification of FD is often challenging given the intentional deception that underpins patient presentation to acute care settings. By Diagnostic and Statistical Manual fifth edition (DSM 5) criteria, factitious disorder imposed on self is characterized by falsified physical or psychological signs or symptoms or induction of disease associated with identified deception and the absence of obvious external rewards for the behavior [4]. Such behavior should not be clearly explained by another mental disorder, and as such care was taken in our case to establish that the patient’s major depressive disorder was indeed well-controlled on a stable antidepressant medication regimen. Although studies of FD historically observed a large proportion of patients to have occupational exposures to healthcare or laboratory settings [3, 5], widespread internet access and availability of medical information online will likely render this association less relevant in modern-day practice. As illustrated by our patient’s explicit reference to pancreatic function, at least some degree of familiarity with the medical approach to hypoglycemia was present at the initial presentation. She also appeared well-adapted to an otherwise dramatic piece of history in volunteering her supposed use of orange juice to treat fasting nocturnal hypoglycemia which awakened her from sleep. Taken together, these statements may represent the application of knowledge gleaned from online sources and her own or her late husband’s prior hospital encounters.

The differential diagnosis of hypoglycemia in adults is broad. Etiologies including sepsis, critical illness with hepatic or renal failure, cortisol deficiency, and drug effects of alcohol should be considered in acutely ill-appearing individuals in the absence of known treatment of diabetes mellitus [2]. For otherwise well-appearing adults, endogenous hyperinsulinism may rarely result from insulinoma, functional pancreatic beta-cell disorders including noninsulinoma pancreatogenous hypoglycemia syndrome (NIPHS) and post-gastric bypass hypoglycemia, or insulin antibody-mediated autoimmune hypoglycemia [2]. Stepwise evaluation of hypoglycemia should first establish whether endogenous insulin production is a driving mechanism. Sample collection must occur during an observed episode of hypoglycemia, and includes measurement of plasma glucose, insulin, C-peptide, and proinsulin concentrations in addition to a screening serum oral hypoglycemic panel [2]. A rise in plasma glucose concentration of at least 25 mg/dL after 1 mg intravenous glucagon administration indicates conserved hepatic glycogen stores [2] and supports endogenous hyperinsulinemia as the principal hypoglycemic process. If spontaneous hypoglycemic episodes cannot be directly observed, a prolonged fast can recreate the circumstances during which hypoglycemia is most likely to ensue [2] and may continue up to 72 hours in coordination with nursing staff to ensure protocol adherence. Fasting C-peptide levels can definitively and reliably differentiate exogenous insulin administration from endogenous hyperinsulinemia. The use of insulin secretagogue agents, however, can result in endogenous hyperinsulinemia biochemically indistinguishable from insulinoma during an episode of fasting hypoglycemia. When endogenous hyperinsulinemic hypoglycemia cannot be attributed to oral hypoglycemic agents, diagnostic procedures to identify and localize an insulinoma are warranted [2].

Factitious hypoglycemia induced by surreptitious insulin or insulin secretagogue administration has long been recognized as a presentation of FD [3]. In our patient, key elements of presentation including a recent pre-diabetes diagnosis and diabetic-range hemoglobin A1c were incongruent with a self-reported history of recurrent hypoglycemia. Although she had previously undergone Roux-en-Y bypass, the remoteness of her surgery (12 years prior) and absence of any postprandial symptom association rendered post-gastric bypass an unlikely etiology. While results of the patient’s initial testing suggested endogenous hyperinsulinemia, the absence of observable hypoglycemia after the first 24 hours of hospitalization, in addition to the practical aspect of required laboratory processing time for screening serum oral hypoglycemic levels, factored into our decision to perform a diagnostic fast. Regardless of the clinical scenario, the occurrence of apparent endogenous hyperinsulinemic hypoglycemia should prompt physicians to consider the possibility of sulfonylurea ingestion (accidental, surreptitious, or malicious) given the widespread availability of insulin secretagogue therapy for diabetes mellitus in the general population, an estimated insulinoma incidence of 1 in 250,000 patient-years [2], and the potential for costly invasive procedures often required to localize such tumors when imaging is equivocal.

Although no reliable estimates of FD prevalence in hospital settings currently exist, most physicians will encounter at least one patient with FD in the course of their careers [3, 6]. Endocrinologists are among the physicians most likely to encounter FD both in outpatient and hospital practice [3, 6]. In one of the largest systematic reviews of FD on 455 published cases from 1965 to 2005, the authors found endocrinology to rank as the medical specialty with the largest total number of FD patient presentations, followed in order of frequency by cardiology and dermatology [3]. For FD cases under endocrinologist purview, recurrent induced hypoglycemia was by far the most common index presentation (31 of 59 cases), ahead of both exogenously induced Cushing’s syndrome (9 of 51 cases) and induced thyrotoxicosis (8 of 51 cases) [3]. Factitious reported chest pain (29 of 44 cases) and induced generalized skin lesions (10 of 43 cases) were the commonest presentations under the care of cardiology and dermatology, respectively [3]. While some have postulated the seemingly high prevalence of induced hypoglycemia observed in published FD cases may represent the relative ease of laboratory diagnosis compared to other subspecialties [3], its recognition is nonetheless critical to preventing profound morbidity and mortality that may otherwise befall FD patients with severe hypoglycemia unless treated in a timely manner.

As with any medical condition, accurate documentation of FD cases in electronic health records is imperative but not without its own specific ethical considerations of which treating physicians should be aware [1]. Importantly, when a patient with multiple chronic medical conditions such as ours (e.g. coronary artery disease, peptic ulcer disease) is evaluated for signs or symptoms deemed factitious in origin after workup, attaching a definitive diagnosis of FD to the patient’s electronic medical record can have unintended consequences of biasing providers faced with evaluating the patient for otherwise concerning organic complaints (e.g. substernal chest tightness or epigastric pain) in the future. The medicolegal implications of missed diagnoses in such a setting also merit strong consideration. Care must therefore be taken to avoid conclusively assigning patients a diagnosis of FD unless documentation also includes ample consideration of alternative differentials and unambiguous evidence to substantiate claims of intentionally deceptive behavior or fabrication. Dismissive or disparaging references to previously unfruitful diagnostic workups during prior hospitalizations for similar complaints must also be avoided. Conversely, when tasked with evaluating patients whose electronic medical records carry a diagnosis of FD or language otherwise suggestive of self-harming healthcare-seeking behavior, physicians need not raise these sensitive historical data on initial encounters unless immediately and inexorably relevant to the clinical circumstances of presentation. When choosing to discuss a diagnosis of FD with affected patients, a method known as “supportive confrontation” [5] should be employed only after meticulous data gathering and through a multidisciplinary approach in conjunction with our psychiatric colleagues [1,5].

## Conclusions

Early detection and management of factitious disorders can prevent the overuse of hospital resources and avoid potential patient harm associated with unnecessary interventions. Both FD and hypoglycemic disorders are uncommon in the general population but for a small subset of patients may overlap in acute care presentation and carry significant risk unless diagnosed and treated in a timely manner. Given widespread insulin secretagogue use for the treatment of diabetes mellitus, physicians should screen for ingestion of these agents with a qualitative serum oral hypoglycemic panel during initial hypoglycemia evaluation in all patients irrespective of medical history. When faced with suspected cases of FD, healthcare providers should be conscientious in electronic medical record documentation and seek assistance from psychiatric services for multidisciplinary care of affected patients.
